# Studying the Salt Dependence of the Binding of σ70 and σ32 to Core RNA Polymerase Using Luminescence Resonance Energy Transfer

**DOI:** 10.1371/journal.pone.0006490

**Published:** 2009-08-03

**Authors:** Bryan T. Glaser, Veit Bergendahl, Larry C. Anthony, Brian Olson, Richard R. Burgess

**Affiliations:** McArdle Laboratory for Cancer Research, University of Wisconsin-Madison, Madison, Wisconsin, United States of America; Baylor College of Medicine, United States of America

## Abstract

The study of protein-protein interactions is becoming increasingly important for understanding the regulation of many cellular processes. The ability to quantify the strength with which two binding partners interact is desirable but the accurate determination of equilibrium binding constants is a difficult process. The use of Luminescence Resonance Energy Transfer (LRET) provides a homogeneous binding assay that can be used for the detection of protein-protein interactions. Previously, we developed an LRET assay to screen for small molecule inhibitors of the interaction of σ70 with theβ' coiled-coil fragment (amino acids 100–309). Here we describe an LRET binding assay used to monitor the interaction of *E. coli* σ70 and σ32 with core RNA polymerase along with the controls to verify the system. This approach generates fluorescently labeled proteins through the random labeling of lysine residues which enables the use of the LRET assay for proteins for which the creation of single cysteine mutants is not feasible. With the LRET binding assay, we are able to show that the interaction of σ70 with core RNAP is much more sensitive to NaCl than to potassium glutamate (KGlu), whereas the σ32 interaction with core RNAP is insensitive to both salts even at concentrations >500 mM. We also find that the interaction of σ32 with core RNAP is stronger than σ70 with core RNAP, under all conditions tested. This work establishes a consistent set of conditions for the comparison of the binding affinities of the *E.coli* sigma factors with core RNA polymerase. The examination of the importance of salt conditions in the binding of these proteins could have implications in both in vitro assay conditions and in vivo function.

## Introduction

The study of protein-protein interactions can often provide great insights into the regulatory mechanisms of cellular pathways. It is desirable to know the strength of the protein-protein interactions, but gathering such information accurately can often be difficult. With the knowledge of the strengths of protein-protein interactions, it is possible to gain insights into competition for binding when multiple proteins interact with the same partner and how the competition could be regulated. There are many ways in which protein-protein interactions can be measured. Non-homogeneous techniques such as surface plasmon resonance [1], pull-down assays (ex. co-immunoprecipitation), enzyme-linked immunosorbent assay (ELISA) [2], size exclusion chromatography [3], and glycerol gradient ultracentrifugation [4] all utilize separation steps that can result in the inaccurate measurements of protein-protein interactions when the transient interactions or weak interactions have half-lives shorter than the time needed for separation to occur. Homogeneous assays such as fluorescence resonance energy transfer (FRET), fluorescence quenching assays [5], [6], luminescence resonance energy transfer/time resolved FRET (LRET/TR-FRET), or fluorescence polarization (FP) all allow for the measurement of protein-protein interactions without the use of a separation step. These assays allow for minimal perturbation of the environment permitting binding to be measured under equilibrium conditions. With either type of binding assay it is important to consider the conditions in which the measurement is made.

FRET and LRET are both assays that measure the energy transfer from a donor fluorophore to an acceptor fluorophore. When the emission spectrum of the donor overlaps with the excitation spectrum of the acceptor resonance energy transfer occurs with a non-radiative energy transfer through dipole-dipole interactions. The magnitude of the resonance energy transfer is distance-dependent, in that the efficiency of energy transfer decreases with the inverse sixth power of the distance between the dyes according to Förster's theory [7]. The fact that the resonance energy transfer is distance dependent makes FRET and LRET highly suitable for the detection and quantification of protein-protein interactions. The key difference between FRET and LRET is that LRET utilizes a lanthanide chelate as the donor instead of a typical organic fluorophore. The lanthanide chelate's emission is technically not fluorescence (i.e. arising from a singlet to singlet transition) and has a very long fluorescent half-live (∼ms) compared to the short half-life (∼ns) of most organic fluorophores. The longer half-life of the lanthanide chelates provides the opportunity to time-gate or delay a measurement after excitation, allowing for reduction of background fluorescence. For a more detailed explanation of LRET, see the following publications [8]–[16].

The *Escherichia coli* transcription machinery is a system in which the protein-protein interactions play a direct role in function. Core RNA polymerase (core RNAP) is a large multisubunit enzyme (α_2_ββ'ω) that is capable of RNA synthesis but is not able to recognize specific promoters [17]. There are seven *E. coli* sigma factors, which have no enzymatic activity, but when bound to core RNAP provide the ability for the newly formed holoenzyme to recognize a unique set of gene promoters and initiate transcription [18], [19]. Therefore it is the interaction between core RNAP and a sigma factor that provides the bacteria the ability to respond to certain stresses by changing the transcription activity of the polymerase.

**Figure 1:**
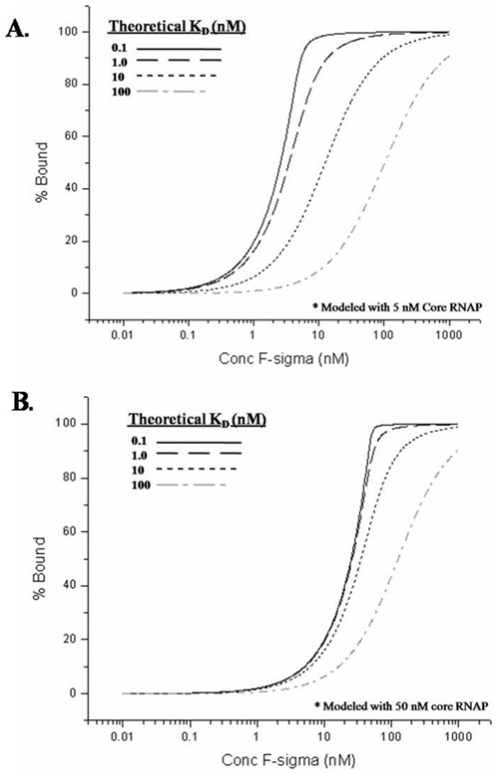
Theoretical binding curves for a sigma factor to core RNAP. Shown is the theoretical modeling of the binding of Tb-core to increasing concentrations of a fluorescein-labeled sigma factor. The X-axis shows the total concentration of F-sigma and the Y-axis is the % core RNAP bound or % holoenzyme formed. The following binding strengths are modeled for: 0.1 nM (solid black line), 1 nM (dashed black line), 10 nM (dotted black line), and 100 nM (dashed-dotted gray line). A) Modeling 5 nM Tb-core. B) Modeling 50 nM Tb-core.

Studying these interactions and what regulates them can provide insights into global transcription regulation. This work focuses on the interaction of σ70 and σ32 with core RNAP. σ70 (RpoD) was the first discovered sigma factor [17] and is the most abundant sigma factor in *E. coli*, responsible for the transcription of genes needed for normal growth. σ32 (RpoH) is the sigma factor that directs the transcripts needed to respond to heat shock and other stress conditions. It has been reported that core RNAP interacts with σ70 with a binding strength of 0.25–0.5 nM [3], [5], [6] as well as 190 nM [1] and σ32 with a binding strength of 1–1.25 nM [3], [4]. Unfortunately, the conditions in which these interactions have been measured were different making it difficult to directly compare the values. We have used our LRET assay to study the strength of binding of σ70 and σ32 with core RNAP and compare the results to those previously published results. In order to measure strong binding constants (K_D_'s in the low nM range) it is important to work in the correct “window of the assay”. It can be seen in Figure 1 that our assay must use concentrations of core RNAP around 5–10 nM and the concentration of sigma is varied (0.1–500 nM) to be able to distinguish tight binding interactions (K_D_∼1 nM). If higher concentrations of core RNAP are used, it will be difficult to distinguish a K_D_ of 1 nM from 10 nM. With the LRET assay we were able to show differential salt sensitivities to NaCl and potassium glutamate (KGlu) for the interaction of σ70 and σ32 with core RNAP. We also show that σ32 interacts more strongly than σ70 with core RNAP in all conditions tested.

## Materials and Methods

### Production and purification of labeled proteins

Sigma factors were produced from *E. coli* BL21(DE3) pLysS carrying pET vectors encoding the appropriate inducible plasmids (LCA57 (pLA4) for σ70 and LN9 (pLHN16) for σ32) as described previously [20]. Briefly, σ70 and σ32 were grown, induced, and inclusion bodies were isolated. Washed inclusion bodies were solubilized in 10 mL of 6 M guanidine-HCl (GuHCl) and centrifuged at 20,000 x g for 15 minutes to remove insoluble material. The solubilized inclusion bodies were refolded by flash dilution with rapid stirring into 600 mL of TGE (50 mM Tris-HCl, pH 7.9, 5% glycerol, 0.5 mM EDTA) plus 15% glycerol and allowed to refold at 22°C for 1 hour. The refolded protein is then passed through a Stericup-GP, 0.22 µm filter (Millipore, Billerica, MA) to remove any particulate material. The filtered solution was then purified using a 15-mL Poros HQ50 (Applied Biosystems, Foster City, CA) anion exchange column pre-equilibrated with TGE. The column was loaded, the protein was eluted at 5 mL/min with a gradient from TGE to TGE+1 M NaCl over 8 column volumes (CV), and 5-mL fractions were collected. The peak fractions (determined by UV absorbance) were collected, pooled, and dialyzed overnight at 4°C into carbonate buffer (100 mM sodium carbonate, pH 9.5, 150 mM NaCl) using a 12-mL Slide-a-lyzer (10,000 Da MW cut off) (Thermo Fisher Scientific, Waltham, MA).

Core RNAP was produced from *E. coli* BL21(DE3) carrying the RLG7651 plasmid for the overexpression of theα, β, β', and ω subunits [21]. An overnight culture was used to inoculate 4×1 L of LB+100 µg/mL ampicillin and 50 µg/mL spectinomycin. The culture was grown at 37°C to an A_600_∼0.45, induced for 3 hours with 1 mM isopropyl β-D-1-thiogalactopyranoside (IPTG), and then harvested by centrifugation at 20,000 x g at 4°C. The resulting pellet (about 10 g wet weight) was resuspended in 50 mL of TE (50 mM Tris-HCl, pH 7.9, 0.1 mM EDTA) plus 5 µL rLysozyme (EMD Biosciences, Gibbstown, NJ) and broken by sonication. The cell debris was cleared by centrifugation at 20,000 x g at 4°C. The core RNA polymerase was purified by polyethyleneimine (PEI) and ammonium sulfate precipitation as described [22]. The resulting ammonium sulfate pellet was resuspended in 40 mL of TE and centrifuged at 20,000 x g at 4°C to remove any insoluble material. The cleared supernatant was loaded onto an 8-mL MonoQ HR 10/10 column (GE Healthcare) equilibrated with TGE. The column was run at 4 mL/min and the protein was eluted with a linear salt gradient of TGE to TGE+0.75 M NaCl over 8 CV. The five peak fractions (10 mL total) were pooled and diluted 15-fold with TGE and loaded onto a 20-mL BioRex70 column equilibrated with TGE. The column was run at 3 mL/min and the protein was eluted with a gradient of TGE to TGE+0.70 M NaCl over 4 CV. The peak fractions (2 mL) were collected and pooled and the BioRex70 purification was repeated to ensure the removal of σ70 [22]. The core RNAP was then dialyzed into carbonate buffer and concentrated for labeling as described below.

Proteins were labeled by diluting the protein to a concentration of 1 mg/mL in the carbonate buffer and incubating with a five-fold molar ratio of dye to protein for 30 min at 22°C as described previously [23]. Two dyes were used for the labeling of the proteins. The selected ratios of fluorescein (Invitrogen, Carlsbad, CA) or CS124-DTPA-Phe-NCS-Tb-chelate (Invitrogen) to protein were used to randomly label lysine residues on the proteins, yielding around 1–2 molecules of covalently bound dye per protein molecule. After labeling, the proteins were diluted ten-fold with TGE. The labeled protein was then loaded onto a 2-mL DE52 DEAE cellulose (GE Healthcare, Piscataway, NJ) gravity column to remove both reactive and hydrolyzed forms of free dye as well as aggregated material. We found it was important to use an inexpensive anion exchange resin for the initial purification because residual reactive dye can chemically attach to the free amines of the column. The column was washed with 3 column volumes of TGE and then eluted with TGE+1 M NaCl. The eluted protein was then purified using either a 120-mL Superdex 200 Prepgrade 16/60 (GE Healthcare) or a 24-mL Superose 6 10/30 (GE Healthcare) size exclusion chromatography column as a final purification step. The columns were equilibrated with TGE+250 mM NaCl and run at 1 mL/min, and 1-mL fractions were collected.

Labeled proteins were examined by SDS-Polyacrylamide gel electrophoresis (SDS-PAGE) to verify purity. Proteins were heated at 75°C for 5 minutes in SDS-samples buffer and then loaded along with the Novex Sharp Pre-Stained Protein Molecular Weight Standard (Invitrogen) onto a 4–12 Bis-Tris NuPAGE gel (Invitrogen) and run at 125 volts. The gel was visualized using a Typhoon Imager (GE Healthcare) to monitor the location of the fluorescein-labeled sigma factor. The gel was then also stained with Gel Code (Thermo Fisher Scientific), a coomassie blue-based stain, to visualize any proteins that did not contain fluorescein.

To store the proteins, the peak fractions (determined by UV absorbance) were pooled and dialyzed overnight at 4°C into storage buffer (250 mM NaCl, 50 mM Tris-HCl, pH 7.9, 0.5 mM EDTA (not used with Tb), 1 mM DTT, 50% glycerol) using a Slide-a-lyzer. The proteins (concentration ∼1 mg/mL) were then stored at −20°C.

### Determination of labeling efficiency

The labeling efficiency was calculated according to manufacturer's instructions. Briefly, the absorbance spectra of the labeled proteins were determined using the Nano Drop ND-1000 Spectrophotometer (Thermo Fisher Scientific). The labeling efficiency was calculated using the following equations:
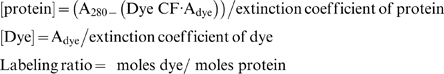



The A_280_ is the absorbance of the protein at 280 nm. The A_dye_ is the absorbance value of the dye at its peak absorbance. The Dye CF is the labeling correction factor provided by supplier of the dye correcting for amount of absorbance at 280 nm from the dye. The molar extinction coefficients (E_280_) used for σ70, σ32, and core RNA polymerase were 39,760 M^−1^ cm^−1^, 43,100 M^−1^ cm^−1^, and 198,500 M^−1^ cm^−1^, respectively (determined from the sequence using Lasergene (DNASTAR, Madison, WI). The molar extinction coefficients used for the CS124-DTPA-Phe-NCS-Tb-chelate (A_345_ and fluorescein (A_495_) were 12,750 M^−1^ cm^−1^ and 77,000 M^−1^ cm^−1^, respectively (according to manufacturer's protocol). The CFs used for CS124-DTPA-Phe-NCS-Tb-chelate and fluorescein were 0.75 and 0.3, respectively.

### Native gel electrophoretic mobility shift assay

The procedure used was modified from the following source [24]. Native gel shift assays using 4–12% Tris-glycine gels (Invitrogen) were performed to assess binding activity of the labeled proteins. 0.8 µg of fluorescein-labeled sigma factor (780 nM final concentration) was incubated alone or in the presence of increasing amounts (0.5–5 µg, 90–900 nM final concentration) of unlabeled core RNAP or Tb-core RNAP for 1 hour in 15 µL of NTG (150 mM NaCl, 50 mM Tris-HCl, 5% glycerol) buffer at pH 8.8. During the incubation, the gel was pre-run at 4°C in Tris-glycine buffer (Invitrogen) at 125 V. The samples were then loaded onto the gel and run for 4 hours at 4°C at 125 V. The gel was visualized using a Typhoon Imager (GE Healthcare) or stained with Gel Code (Thermo Fisher Scientific), as described above.

### Effect of buffer components on LRET assay

Standard components of buffers used for transcription or drug screening were tested for their effect on the binding of 10 nM Tb-core to 20 nM fluorescein-σ70 (F-σ70). The following components were tested: DMSO, ethanol, methanol, TritonX-100 (Surfact-Amps X-100, Thermo Fisher Scientific), Tween-20 (Surfact-Amps 20, Thermo Fisher Scientific), bovine serum albumin (BSA), MgCl_2_, and glycerol. Triplicate serial dilutions of a 2X stock of each component were made using MilliQ dH_2_O (15 µL final volume) in black, flat-bottom polystyrene NBS 384-well microplates (Corning, Corning, NY). To measure the LRET, 15 µL of a 2X complex (20 nM Tb-core, 40 nM F-σ70) in 2X TG (100 mM Tris-HCl, pH 7.9, 10% glycerol)+200 mM NaCl was added to 15 µL of a 2X serial dilution of the various components, mixed up and down five times with a pipette (30 µL total volume, final concentration of 10 nM Tb-core/20 nM F-sigma in TG+100 mM NaCl) and then incubated covered for 1 hour at 22°C (room temperature). The 2X complex was made by diluting the labeled proteins from the storage buffer (-20°C) directly into the TG+100 mM NaCl buffer at 22°C. After the incubation, a VictorV® Plate Reader (Perkin Elmer, Waltham, MA) was used to measure the LRET signal. The samples were excited with 1,000 flashes at 340 nm and measurements were delayed for 100 µs to allow time for decay of background fluorescence. The data were acquired for 200 µs at 490 nm (Tb signal) and 520 nm (fluorescein signal). The average ratio of the acceptor signal to the donor signal (A_520_/A_490_) for each triplicate was calculated according to Riddle *et al*. [25]. The average of the acceptor to donor signal is used to correct for any differences in donor signal. It is also useful for correcting for some occurrences of quenching or light scattering that would be encountered with a high throughput screen of small molecules.

### Effect of salt on LRET assay

Salt dose curves were performed with the LRET assay to determine the effect of salt type and concentration on the sigma-core interaction. 15 µL of a 2X serial dilution of either 1 M NaCl or 1 M KGlu in TG (50 mM Tris-HCl pH 7.9, 5% glycerol) was performed in quadruplicate in black, flat-bottom polystyrene NBS 384-well microplates. 15 µL of a 2X complex of 10 nM Tb-core/20 nM fluorescein-sigma (final concentration) was added to the NaCl and KGlu serial dilutions, mixed and then incubated for 1 hour at 22°C. The salt contributed by the proteins was negligible. The assay was performed and analyzed as above.

### LRET binding strength assay

A saturation binding assay was performed to determine the strength of the interaction of F-σ70 and fluorescein-σ32 (F-σ32) with Tb-core RNAP. A 2X serial dilution of the fluorescein-sigma was made in TG plus 100 mM, 250 mM, or 500 mM NaCl or KGlu. A 2X solution of 10 nM Tb-core RNAP (final concentration) in TG with the corresponding salt was added and mixed. The samples were incubated for 1 hour at 22°C and the LRET was measured as described above. The lowest average A/D ratio for each set of data was subtracted from the data set to create a similar baseline for each data set (background was 5-10% of the maximum signal). The data were fit using Origin 7 (OriginLab, Northampton, MA). The following binding model was used to determine the strength of binding:
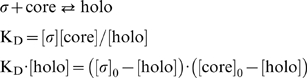
This can be rearranged to:
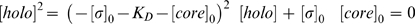
Solving the quadratic equation results in: 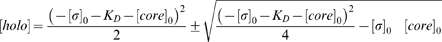
[Holo] is the concentration of holoenzyme as measured by the LRET signal (A/D ratio), [σ]_0_ is the total concentration of sigma used, and [core]_0_ is the total concentration of core used in the assay. A nonlinear least squares fitting of the data using Levenberg-Marquardt iterations was used to analyze the data. The program was allowed to determine the best values for K_D_. To achieve optimum fitting as determined by Chi-squared and R^2^ values, the value for [core]_0_ was set at 5 nM instead of 10 nM. The lowering of the concentration of Tb-core was supported by the lowered binding activity (∼50% active) observed in the gel shift assay. The maximum binding (A/D ratio) was set at the value where saturation was reached (1.2 for σ70 and 1.6 for σ32). The saturation levels for σ70 and σ32 were different due to the different amount of fluorescein-labels on each protein. The setting of this value was done under the assumption that if all samples contain the same amount of core, they should saturate binding at the same level. The assumption was necessary to fit the data that did not reach saturation.

## Results

### Protein labeling, purification, and characterization

**Figure 2:**
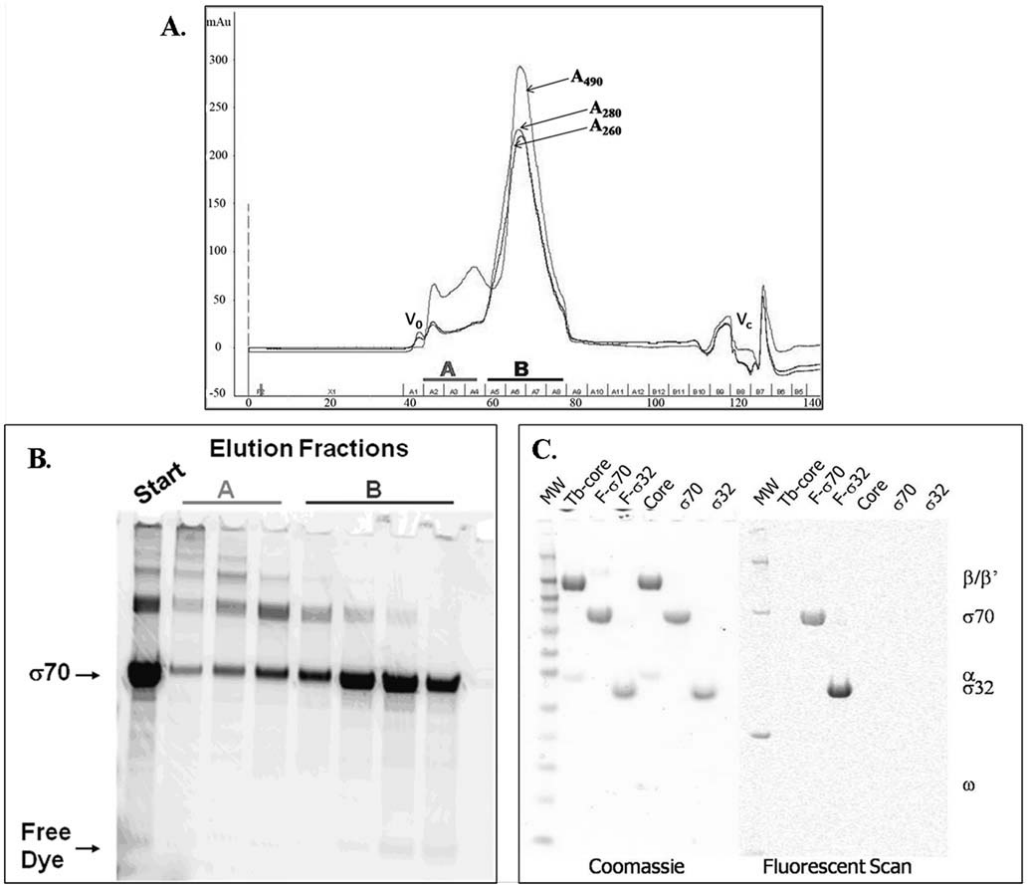
An overview of the labeling and purification procedure. A two-step purification procedure was used to purify the proteins after being labeled with either fluorescein or a terbium chelate. The completed labeling reaction was first purified using a DEAE cellulose gravity flow column and the peak fraction was further purified using size exclusion chromatography A) A Superdex 200 Prepgrade 16/60 size exclusion chromatography column is used to separate the aggregates as well as residual free dye from the labeled protein. Three absorbance readings are monitored on the chromatogram, A_260_, A_280_, and A_490_ to measure fluorescein absorbance. V_0_ indicates the void volume and V_c_ indicates the column volume. Peak A and peak B both contain fluorescein-labeled σ70. B) A fluorescent scan of a 4–12% SDS-PAGE showing peak A and peak B. Peak B was pooled and used for binding studies. C) An SDS-PAGE of the purified proteins used in the LRET assays. The coomassie stain and fluorescent scan using the Typhoon Imager of 5 µg of the labeled and unlabeled proteins are shown. Note that the 260, 80, and 20 kDa markers (Novex Sharp Pre-Stained Protein Molecular Weight Standard) are fluorescent.

The preparation of the labeled proteins used in the LRET assay is very important for the accuracy of the future experiments. In most cases the addition of a fluorescent dye results in the addition of a small hydrophobic patch on the protein of interest. This addition of hydrophobicity could theoretically alter the binding properties of the protein and appropriate controls need to be carried out. The proteins (1 mg/mL concentration) were labeled for 30 minutes at 22°C with a 5-fold molar ratio of dye to protein at pH 9.5. These conditions minimize over-labeling of the protein, typically yielding 1–2 molecules of dye per protein molecule depending on the protein used [23]. There are 34 lysine residues (5.5%) in σ70, 16 lysine residues in σ32 (5.6%), and 202 lysine residues in core RNAP (5.8%). Once the protein has been labeled, it is critical to remove any free label and/or multimerized protein that may have resulted. Free label can cause an increase in background signal due to diffusion-limited LRET and multimers can decrease the activity of the protein population. Diffusion-limited LRET is observed when the concentration of either the free dye or the labeled proteins increase to a level where a false-positive signal occurs when the donor and acceptor are in close proximity while passing by diffusion and not through binding. Two purification steps are used to clean up the protein sample. First the sample is diluted 10-fold with TGE and purified by gravity anion-exchange chromatography using DE52 DEAE cellulose resin to concentrate the sample while also removing the majority of the free dye. The concentrated protein is then loaded onto a size-exclusion chromatography column to remove the remainder of the free dye. Figure 2 shows the removal of the free dye from the sample as well as the separation of multimer forms of the protein from monomer as visualized with a fluorescent scan of the SDS-PAGE using a Typhoon imager. The final protein is then dialyzed into storage buffer and stored at −20°C.

Once the protein has been labeled and purified, it needs to be characterized for labeling efficiency, concentration, and to determine if the labeling has altered the activity. The protein concentration and labeling efficiency was determined by spectrophotometry. Core RNAP was labeled with a 1∶1 ratio of Tb-chelate to molecule of protein and stored at 2.7 µM (1.1 mg/mL). σ70 and σ32 were labeled with a 1.4∶1 and 2∶1 ratio of fluorescein to molecule of protein, respectively. σ70 and σ32 were stored at 23.4 µM (1.4 mg/mL) and 28.7 µM (0.92 mg/mL), respectively.

**Figure 3:**
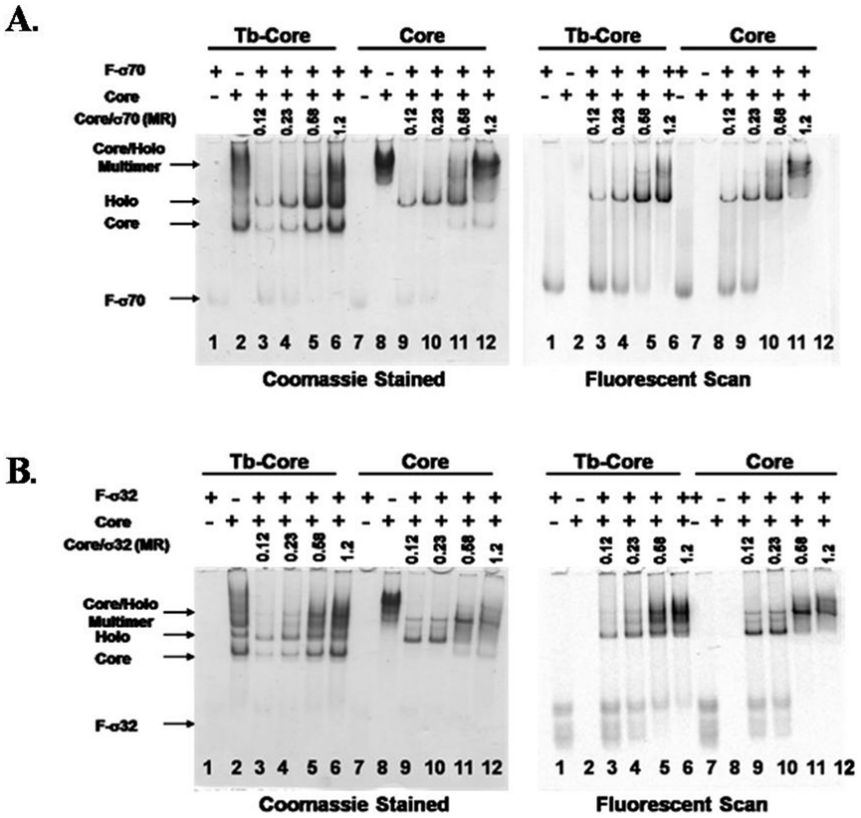
Electrophoretic mobility shift assay to determine quality of labeled proteins. An electrophoretic mobility shift assay was used to determine if all of the fluorescein-labeled sigma factors were able to form holoenzyme and if the terbium-labeled core could bind fluorescein-sigma as well as unlabeled core. Proteins were incubated for 1 hr at 22°C and run on a native 4-12% Tris-glycine gel. The F-sigma factors were visualized using the Typhoon Imager to produce a fluorescent scan of the gel (right). The total protein was visualized by staining the gel with Coomassie stain (left). The molar ratio (MR) of core to sigma is indicated. A) F –σ70 (780 nM final concentration) was incubated with increasing concentrations of Tb-core or unlabeled core (90–900 nM final concentration). B) F –σ32 (780 nM final concentration) was incubated with increasing concentrations of Tb-core or unlabeled core (90–900 nM final concentration).

Electrophoretic mobility shift assays were performed to determine if the labeling altered the ability of the proteins to interact. This assay can determine if all of the labeled sigma factor is able to bind to core RNAP and if terbium-labeled core is able to bind to sigma as well as unlabeled core. The assay shows that both F-σ70 and F-σ32 are able to be shifted completely to holo by the addition of core RNAP and therefore are 100% active in binding. The assay also shows that the terbium-labeled core is slightly less active in its ability to shift both F-σ70 and F-σ32 as compared to unlabeled core (Figure 3). This inactive labeled core could be caused by the labeling of the core RNAP if some of the dye derivatized lysines prevent the interaction with sigma. It appears that the Tb-labeled core contains a population (35–50%) that is inactive in binding sigma. Similar results have been obtained with many other batches of proteins utilizing a variety of fluorescent labels (data not shown for proteins labeled with Alexa Fluor546, Alexa Fluor 555, or Alexa Fluor647 (Invitrogen)). The activity can vary from batch to batch even with unlabeled proteins, due to steps in the production such as protein refolding. The decreased activity was accounted for when the data were fit as described above. It can also be seen that some of the labeled core RNAP tends to run at a faster mobility than the unlabeled core RNAP during non-denaturing gel electrophoresis, suggesting that the unlabeled core RNAP has a somewhat greater tendency to form higher multimers. The F-σ32 also contained two species that were both able to interact with core RNAP to form holoenzyme. It is most likely that the purified F-σ32 and unlabeled-σ32 contained some dimer or higher multimers that are not evident in the denaturing SDS-PAGE (Figure 2C).

### The effect of buffer components on the sigma-core RNAP interaction

**Figure 4:**
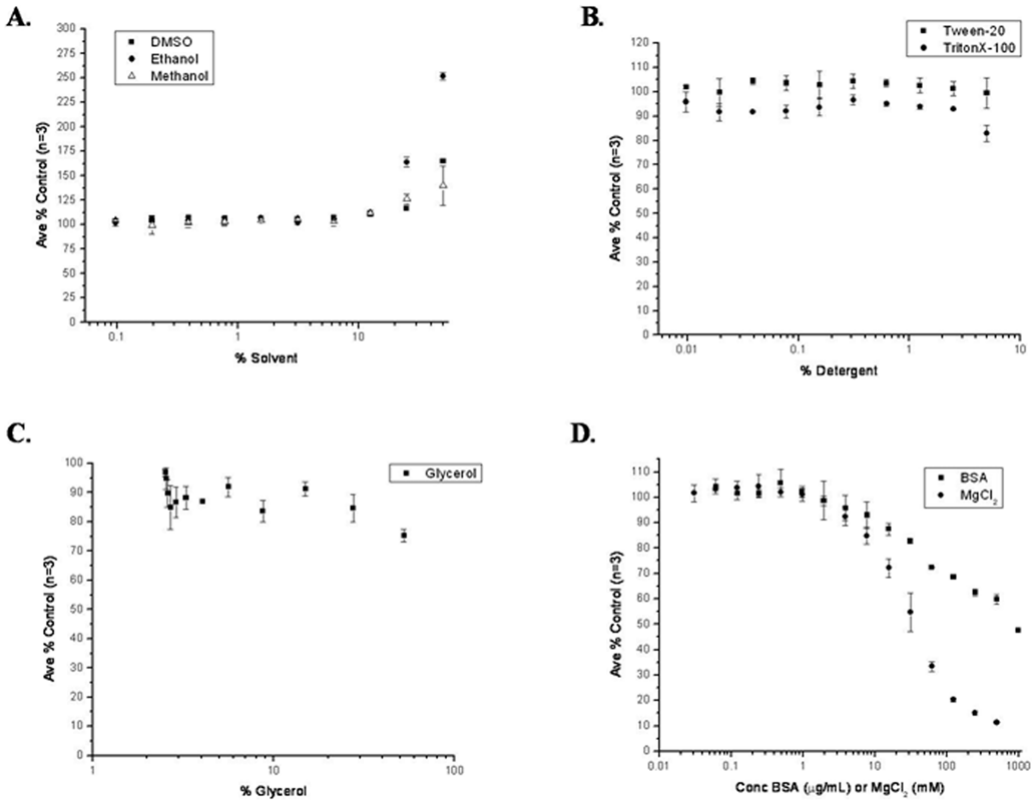
The effect of common buffer components on the LRET assay. Common buffer components were tested for their ability to alter the interaction 10 nM Tb-core with 20 nM F-σ70 after a 1-hour incubation at 22°C. The average value of A_520_/A_490_ for three replicates was determined and normalized to a no-treatment control with a buffer containing 50 mM Tris-HCl pH 7.9, 5% glycerol, and 100 mM NaCl. The error bars represent the normalized standard deviation for a sample size of 4. A) Common solvents (DMSO, ethanol, and methanol) were tested in a range of 0–50% (v/v). B) The non-ionic detergents TritonX-100 and Tween-20 were tested from 0–5% (v/v). C) Glycerol was tested from 2.5–50% (v/v). The minimum glycerol level (2.5%) was due to the glycerol in the buffer of the proteins. D) Bovine serum albumin (BSA) and MgCl_2_ were tested from 0–1000 µg/mL and 0–500 mM, respectively.

The LRET assay was used to determine how common buffer components affect the binding of 10 nM Tb-core to 20 nM F-σ70. Common solvents such as DMSO, methanol, and ethanol had no effect on the binding at percentages (v/v) up to 10% (Figure 4A). Binding was seen to increase at higher percentages, perhaps due to non-specific binding after denaturation. Non-ionic detergents such as TritonX-100 and Tween-20 had no effect up to 2.5% (Figure 4B). Glycerol also had very little effect on the binding up to 55% (Figure 4C). Two components, BSA and MgCl_2_, were found to have inhibitory effects at higher concentrations (Figure 4D). BSA had no effect at concentrations up to 10 µg/mL (150 nM), but inhibited roughly 50% of binding by 1 mg/mL (15 µM). MgCl_2_ had an inhibitory effect with an IC_50_ around 30 mM. It is known that Mg^2+^ can cause the disruption of protein binding to DNA, by weakening ionic interactions [26]. It is possible a similar thing is happening with the interaction of F-σ70 with Tb-core RNAP. It is also possible that the Mg^2+^ is promoting the multimerization of core RNAP as reported in [27], reducing its ability to bind sigma.

**Figure 5:**
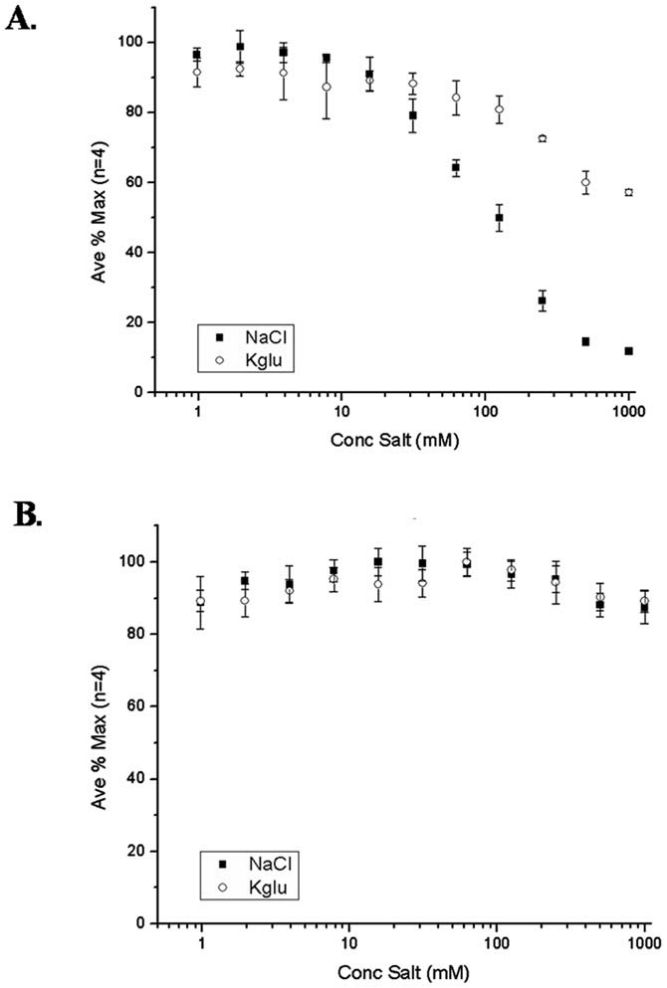
Salt dependence of the sigma-core interaction. An LRET assay was performed to determine the effect of NaCl or KGlu on the binding interaction of 10 nM Tb-core with either 20 nM F-σ70 or F-σ32. Shown is the average acceptor/donor signal (A_520_/A_490_) for each sample normalized to a sample with the maximum signal. The error bars represent the normalized standard deviation for a sample size of 4. NaCl samples are represented with a solid square and KGlu is represented with an open circle. A) The effect of NaCl or KGlu on the interaction of F-σ70 with Tb-core. B) The effect of NaCl or KGlu on the interaction of F-σ32 with Tb-core.

The LRET assay was performed to determine how salt type and concentration can affect the ability of either σ70 or σ32 to interact with core RNAP. NaCl was chosen due to its prevalent use in most *in vitro* assays and potassium glutamate (KGlu) was chosen because it is the major physiological salt in most *E. coli* cellular conditions [28], [29]. The effect of the two salts was tested by incubating a complex of 10 nM Tb-core with 20 nM F-σ70 or F-σ32 and measuring the resulting LRET signal. It was hypothesized that the binding would decrease at high salt concentrations due to a weakening of the ionic contribution to binding [30]. It was found that F-σ70 binding to Tb-core was very sensitive to NaCl but not as sensitive to KGlu (Figure 5A). In comparison, F-σ32 binding to Tb-core was not sensitive to either NaCl or KGlu even at concentrations reaching 1 M (Figure 5B). These results suggest that the salt type used can cause a dramatic difference in the level of binding between two proteins. It also suggests that the interaction of σ70 and σ32 with core RNAP may differ in the interactions that provide the majority of their binding energy.

### The effect of salt on binding strength

**Figure 6:**
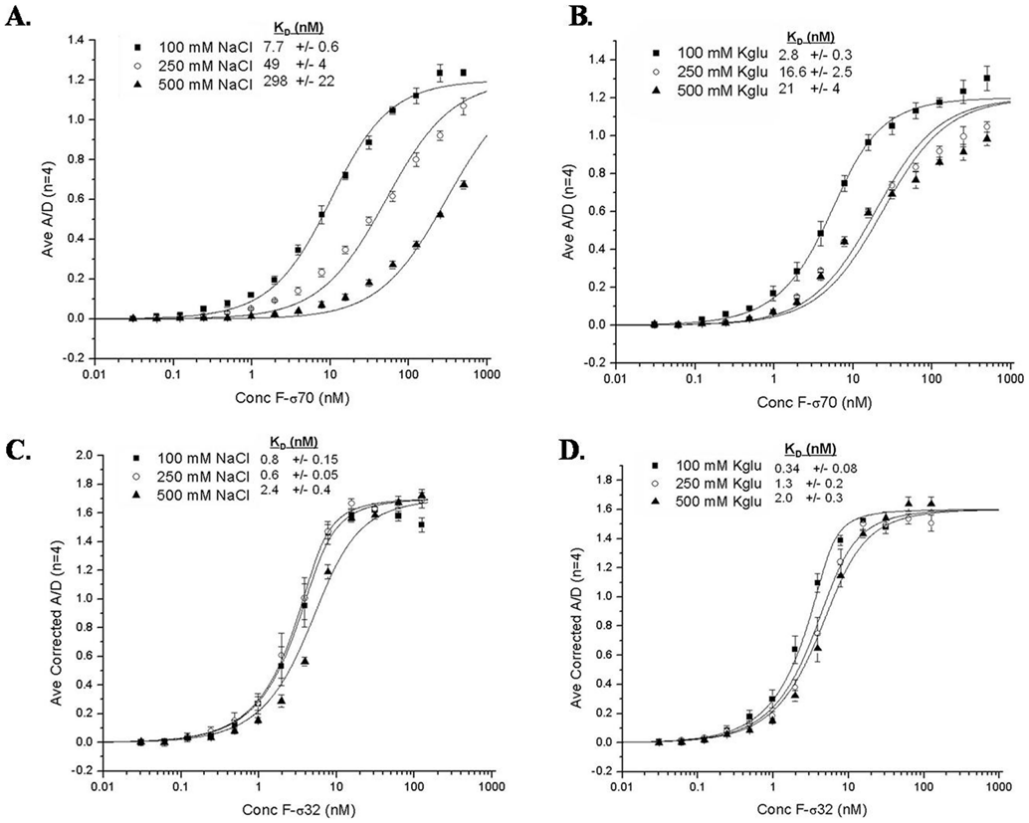
The salt dependence of the interaction of F-σ70 or F-σ32 with 10 nM Tb-core. To determine the effect of NaCl and KGlu on the interaction strength, binding curves were generated for the interaction of either F-σ70 or F-σ32 with 10 nM Tb-core at 100 mM, 250 mM, or 500 mM NaCl or KGlu. Shown is the average acceptor/donor signal (A_520_/A_490_) for each sample. The error bars represent the standard deviation for a sample size of 4. The curves were fit with Origin 7 and the K_D_ values are listed for each curve. A) The effect of NaCl on the interaction of F-σ70 with 10 nM Tb-core. B) The effect of KGlu on the interaction of F-σ70 with 10 nM Tb-core. C) The effect of NaCl on the interaction of F-σ32 with 10 nM Tb-core. D) The effect of KGlu on the interaction of F-σ32 with 10 nM Tb-core.

An LRET saturation binding assay was used to determine the strength of F-σ70 and F-σ32 interaction with Tb-core at 100 mM, 250 mM, and 500 mM NaCl or KGlu. The assay was performed by incubating increasing concentrations of the fluorescein-labeled sigma factor with 10 nM Tb-core in a TG buffer with the desired salt condition for 1 hour at 22°C. It was found, as predicted above, that the F-σ70 interaction with Tb-core was extremely sensitive to NaCl with the equilibrium binding strength (K_D_) dropping from 7.7 nM to 49 nM to 298 nM when increasing the concentration of NaCl from 100 mM to 250 mM to 500 mM, respectively (Figure 6A). The exact binding strength at 250 mM and 500 mM NaCl may be inaccurate, due to the fact that binding was much weaker and saturation could not be achieved in this assay. The effect of KGlu on the interaction of F-σ70 with Tb-core was much less dramatic than that of NaCl, with K_D_'s of 2.8 nM, 16.6 nM, and 21 nM at 100 mM, 250 mM and 500 mM KGlu, respectively (Figure 6B). The prediction (from Figure 5B) of a minimal salt effect on the binding of F-σ32 to 10 nM Tb-core was also accurate. When F-σ32 was tested in NaCl, the binding strength decreased from 0.8 nM to 0.6 nM to 2.4 nM in 100 mM, 250 mM, and 500 mM NaCl, respectively (Figure 6C). The binding in KGlu decreased from 0.34 nM to 1.3 nM to 2.0 nM in 100 mM, 250 mM, and 500 mM KGlu, respectively (Figure 6D). These data confirm that σ70 and σ32 have different salt sensitivities in their interaction with core RNAP.

## Discussion

### Characterization of the LRET binding assay

We have described a simple homogeneous binding assay based on luminescence resonance energy transfer. This assay is a proximity assay that measures the resonance energy transfer from a terbium-chelate donor to a fluorescein-labeled acceptor as described previously [25]. This assay was used for the study of the interaction of the *E. coli* σ70 and σ32 with core RNAP but could be easily extended to any two interacting proteins. Similar assays, utilizing theβ'coiled-coil fragment (amino acids 100–309) have been used in our lab to screen for small molecule inhibitors of the interaction of σ70 with core RNAP [31], [32]. In the previous assay, site-specific labeling at cysteine residues was used to create the LRET pair. In this work we have used random labeling of lysine residues to label core RNAP and sigma factors. This approach is different from the past assay because the random labeling allows the use of proteins for which the creation of single cysteine mutants is not feasible.

Before using the assay to monitor the interactions of the sigma factors with core RNAP, it was important to fully characterize the assay components and understand the assumptions that are made when using the assay. The first important consideration is the properties of the labeled proteins. We make the assumption that the labeling process does not change the binding activity of the proteins. The first control for this assumption is the production and purification of the labeled proteins. The proteins are labeled in a manner that attached 1-2 labels per molecule of protein. Often the dyes being attached are hydrophobic and if too many are attached it is reasonable that the binding properties of the protein could be altered. By limiting the number of labels per protein, the chance of altering the activity is minimized. The purification scheme is also designed to remove all excess non-reacted label that may cause background in the assay as well as any protein multimers formed because of the labeling process. It is extremely important to test all labeled proteins to determine if the activity has been altered. The native electrophoretic mobility shift assay was used to determine if the label has changed the ability of sigma and core RNAP to interact (Figure 3). Analysis of this binding shows that both F-σ70 and F-σ32 are shifted 100% to the holoenzyme when core RNAP is added in excess. The analysis also shows that the Tb-core may be slightly less active in binding sigma than the unlabeled core. The labeled proteins could also be tested for activity in assays such as *in vitro* transcription and promoter binding, but for the context of these experiments it was most important to understand the effect of labeling on the protein binding properties. Ultimately, the activity that will be measured for any labeled protein should be compared to the activity of the unlabeled protein.

### Site-specific versus random labeling of proteins

Site-specific mutations are often used in LRET or FRET to direct the addition of one label to a certain site in a structure by moving the location of one cysteine. The ability to label a protein at a specific site allows for the creation of a homogeneous population of the labeled protein. This labeling technique allows for the elimination of over-labeling the protein and is necessary when using FRET to monitor conformational changes [9]. Unfortunately, not all proteins can be created with a single cysteine. Sometimes the large number of cysteines that need to be removed or the importance of specific cysteines for the structure or activity of the protein makes site-specific labeling of the protein impossible. We used random labeling of lysine residues to avoid these specific problems when using core RNA polymerase which contains 36 cysteine residues. By controlling the labeling conditions to yield 1–2 molecules of label per protein we can create a population of labeled protein that should contain at least one dye within the Förster radius (∼65 Å for this dye pair) allowing for efficient energy transfer. Examination of the structure of *Thermus thermophilus* holoenzyme [33] shows that the vast majority of lysines in sigma and core RNAP are within 65 Å and therefore most of the population of labeled proteins is capable of participating in LRET (data not shown).

### The effect of common buffer components

Other assumptions that could influence the accuracy of binding data are the assumptions that proteins, labeled or not, act consistently over time and from batch-to-batch. We also make the assumption that most buffer components do not affect the interaction that is being studied. This assumption can be tested by isolating single buffer components and testing a concentration range against the normal buffer conditions. Figure 4 demonstrates how components such as solvents, detergents, and other additives affect the binding of σ70 to core RNAP. It was found that the described LRET assay is very robust in that it can tolerate common solvents up to around 8% as well as non-ionic detergents up to around 5%. Similar results have been observed for an LRET assay examining the interaction of σ70 with a fragment of core RNAP [31]. It was also found that additives such as BSA and MgCl_2_ can weaken the interaction of σ70 with core RNAP, depending on the concentration used. It is not clear if these effects are important for physiological interactions but need to be controlled for if used in this assay. We did not use BSA or MgCl_2_ in our binding assay. The effect of temperature on K_D_ was tested (1 hour incubation at 22°C, 37°C, and 45°C), but no difference was measured (data not shown).

### The window of the assay

It is important to also consider that binding assays have a particular range in which it is possible to measure binding strengths accurately. This “window of the assay” must be considered when an experiment is being designed. Figure 1A shows theoretical binding curves that would be generated if a sigma factor interacted with 5 nM core RNAP with a K_D_ of 0.1, 1, 10, or 100 nM. Figure 1B shows the same binding strengths modeled with 50 nM core RNAP. When using the increased core concentration, it is almost impossible to distinguish the 0.1 nM curve from the 1 nM curve. If too much core is used to perform the assay, it will be difficult to distinguish strong interactions. The lower limit (∼1 nM donor) that can be measured with the assay is determined by the signal strength of the LRET pair. The upper boundary of the assay (∼250–500 nM acceptor) is determined by the concentrations at which diffusion-limited LRET begins to create a false-positive for binding. The lower limit of the assay is low nM depending on the number of labels per protein.

### The effect of salt on the interaction of σ70 and σ32 with core RNAP

The LRET assay was used to determine the optimal salt type and concentration to use when studying the interaction of 20 nM F-σ70 or 20 nM F-σ32 with 10 nM Tb-core RNAP (Figure 5). It was observed that the binding of F-σ70 to Tb-core RNAP was much more sensitive to NaCl than was σ32. NaCl caused the weakening of the F-σ70-core interaction whereas F-σ32 was able to remain fully bound at concentrations up to 1 M. It was also found that F-σ70 was much more resistant to KGlu, whereas F-σ32 again did not have any decrease in binding. This differential salt effect suggests that F-σ70 and F-σ32 have somewhat different binding mechanisms with core RNAP. When comparing binding data it is important to consider the buffer conditions in which the interactions were measured because conditions such as salt type and concentration can significantly alter the interaction.

The LRET assay was used to determine equilibrium binding constants for the interaction of F-σ70 and F-σ32 with core RNAP. It was found that the binding of F-σ70 to Tb-core RNAP was variable depending on salt concentration and type. The binding affinity significantly weakened as the concentration of NaCl was increased. It should be noted that the determination of binding constant is less accurate at the higher NaCl because saturation could not be achieved in the assay, but it is clear that binding is significantly weakened. The prediction that KGlu would not affect the interaction of F-σ70 with Tb-core was also confirmed. The binding in the presence of KGlu was generally stronger for σ70 and did not decrease as much at the higher concentrations. The affect of salt on the binding of F-σ32 to Tb-core RNAP was confirmed; the binding was very similar with NaCl or KGlu and the binding strength was not greatly weakened, even at 500 mM.

### Comparison of binding data

The values for the binding of σ70 and σ32 to core RNA polymerase are similar to many of the values obtained in previous studies (Table 1). Unlike the previous values, our set of data can be directly compared because of the consistent set of conditions used. These same conditions could be repeated with the remaining sigma factors to allow for a more detailed comparison of binding strengths. It is difficult to directly analyze the previous results because the effect of salt and other buffer components, as well as the high concentrations of proteins used, can directly change or decrease the accuracy of the measured binding affinity. In particular, we question whether K_D_'s of around 1 nM could possibly be measured using concentrations greater than 100 nM core RNAP, given the theoretical curves presented in Figure 1b. Our results provided extra value because the experiments and controls were carefully performed with concentrations of proteins that can accurately generate K_D_ values in the range we described. We acknowledge that uncertainties in protein concentration, activity, and the fit of the model can also change the affinity measured but the relative order of magnitude of the binding affinities is clear. It is not apparent if the difference between a 1 nM and 2 nM binding strength is significant due to all of the uncertainties mentioned. It is important to recognize that these same problems and uncertainties are present for all binding assays.

**Table 1:** Comparison of binding affinities (K_D_'s).

Sigma	Method	Max [Core] (nM)	Salt	[Salt] (mM)	K_D_ (nM)
σ70					
	FRET^1^	?	KCl	250	≥1
	HPLC gel filtration^1^	>1000	KCl	250	≥10
	FRET^2^	40	KCl	200	0.3
	SPR^3^	490	NaCl	150	190
	SEC^4^	400	NaCl	200	0.3
	LRET	10	NaCl	100	8
			NaCl	250	50
			NaCl	500	300
			Kglu	100	3
			Kglu	250	17
			Kglu	500	21
σ32					
	SEC^4^	400	NaCl	200	1.24
	*in vitro* transcription 5	100	NaCl	100	1
	LRET	10	NaCl	100	0.8
			NaCl	250	0.6
			NaCl	500	2.4
			Kglu	100	0.3
			Kglu	250	1.3
			Kglu	500	2

1 Binding studies performed by Gill *et al.* (1991).

2 Binding studies performed by Wu *et al.* (1976).

3 Surface plasmon resonance studies performed by Ferguson *et al.* (2000).

4 Size exclusion chromatography studies performed by Maeda *et al.* (2000).

5 *In vitro* transcription studies performed by Joo *et al.* (1997).

It can be concluded, under all of the conditions we tested, that the binding of σ32 was determined to be stronger than σ70. The two sigma factors also had very different responses to salt which could suggest differences in the way they interact with core RNAP. The concentrations of all the proteins used are comparable to the amount used in most *in vitro* transcription reactions but are significantly less than the concentrations (µM range) of the proteins in the cell [34], [35]. The measured tight binding of σ70 and σ32 to core RNAP could be significant for interactions in the cell because the high affinity along with the high concentration of the proteins in the cell makes it difficult to imagine much free sigma or core RNAP, except after sigma release during transcription and core RNAP release upon transcription termination.
